# Identification of Peptide Antagonists to Thioredoxin Glutathione Reductase of* Schistosoma japonicum*

**DOI:** 10.1155/2018/9483928

**Published:** 2018-06-05

**Authors:** Li-Jun Song, Jia-Huang Li, Xu-Ren Yin, Wei Zhang, Yi Jin, Hong Gao, Jie Wang, Chuan-Xin Yu, Zi-Chun Hua

**Affiliations:** ^1^School of Life Sciences and the State Key Laboratory of Pharmaceutical Biotechnology, Nanjing University, Nanjing, Jiangsu Province 210023, China; ^2^Key Laboratory of National Health and Family Planning Commission on Parasitic Disease Control and Prevention, Jiangsu Provincial Key Laboratory on Parasite and Vector Control Technology, Jiangsu Institute of Parasitic Diseases, Wuxi, Jiangsu Province 214064, China; ^3^Changzhou High-Tech Research Institute of Nanjing University and Jiangsu Target Pharma Laboratories Inc., Changzhou, Jiangsu Province 213164, China; ^4^Department of Pathology, Nanjing Drum Tower Hospital, The Affiliated Hospital of Nanjing University Medical School, Nanjing, Jiangsu Province 210008, China; ^5^Shenzhen Research Institute of Nanjing University, Shenzhen, Guangdong Province 518057, China

## Abstract

Schistosomiasis is one of the world's major public health problems. Praziquantel is currently the only effective drug against schistosomiasis. As resistance of praziquantel has emerged in some endemic areas, development of new antischistosomal agents should be a high priority. In this study, a phage display peptide library was used for screening for peptide antagonists of thioredoxin glutathione reductase of* Schistosoma japonicum* (SjTGR), which has been identified as an alternative drug target. Three rounds of panning produced four different fusion phages. ELISA proved that all four phages could bind to SjTGR. One peptide, JIPDys1 (aa, WPHNWWPHFKVK), reduced enzyme activity of SjTGR by more than 50%. 2 *μ*M of the synthesized peptide of JIPDys1 inhibited the activity of TrxR, GR, and Grx of SjTGR by 32.5%, 100%, and 100%, respectively. The IC_50_ values of the synthetic peptide JIPDys1 for TrxR, GR, and Grx were 3.67 *μ*M, 0.11 *μ*M, and 0.97 *μ*M, respectively. Based on computer simulation, it appeared that JIPDys1 binds to the substrate binding sites of glutathione reductase (GR) and glutaredoxin (Grx). Our data show that the peptide, JIPDys1 (aa, WPHNWWPHFKVK), is a promising candidate to develop novel drugs against* S. japonicum* which acts by binding with SjTGR and reduces enzyme activity of SjTGR.

## 1. Introduction

Schistosomiasis, a serious disease caused by intravascular trematodes of the genus* Schistosoma*, is one of the world's major public health problems [1–4]. Adult parasites reside in the mesenteric veins of their human hosts, surviving on the host's red blood cells, which results in anemia, diarrhea, splenomegaly, liver fibrosis, and other symptoms [5]. The drug of choice, praziquantel (PZQ) [6, 7], is administered globally to 100 million people each year; but sensitivity problems are emerging. Decreased sensitivity of* Schistosoma mansoni* and* Schistosoma haematobium* to praziquantel has been reported in some endemic areas [8–14]. Artemisinin, which was developed as antimalaria drug, seems to be active against* Schistosoma *[15]. However, juvenile worms are more sensitive to the drug than adult worms [15]. As there is currently no other effective treatment against schistosomiasis, development of new antischistosomal agents to curb the emergence of drug-resistance should be a high priority.

Organisms are continuously attacked by reactive oxygen species (ROS). There are two major systems existing to detoxify ROS in eukaryotes, the thioredoxin (Trx) system, and the glutathione (GSH) system, depending on the Trx reductase (TrxR) and GSH reductase (GR), respectively [16]. However, it has been reported that thioredoxin glutathione reductase (TGR) in plathelminthes such as* S. mansoni *[17–20],* S. japonicum *[21],* larval Taenia crassiceps* (cysticerci) [22],* Echinococcus granulosus* [23], and* Fasciola hepatica *[24] has replaced the separate enzymes TrxR and GR and plays a critical role in thiol-disulfide redox homeostasis. Our initial work using recombinant* S. japonicum *TGR [21] suggested that TGR was essential for* S. japonicum* survival and suited as potential target for development of novel drugs against* S. japonicum*.

Techniques using library screening have improved other methods [19, 25] to identify components as potential drugs against diseases. These new methods have hugely increased speed and throughput to identify interesting lead candidates. In the present study, one of such methods, phage display peptide library screening, was used to identify peptide antagonists to SjTGR. Computer simulation and molecular docking were used to analyze interacting sites of the phage display peptide with SjTGR. The results show that a novel peptide inhibited SjTGR activity likely by preventing the binding of substrates to SjTGR. Our findings lay the foundation for the further development of peptide drugs against the activity of SjTGR.

## 2. Materials and Methods

### 2.1. Phage Display Peptide Library Screening with Recombinant SjTGR

The purchased Ph.D-12 phage display peptide library kit (New England Biolabs, Massachusetts, USA) contained 1x10^13^ pfu/ml with a complexity of 1.0x10^12^ transformants and used filamentous coliphage M13 for peptide expression. Phage was propagated in* Escherichia coli* strain ER2738 provided with the kit. 3,3',5,5'-Tetramethylbenzidine (TMB) substrate solution was purchased from Neobioscience Technology Company Limited (Beijing, China) and horseradish peroxidase (HRP) conjugated anti-M13 monoclonal antibody from GE Healthcare Life Sciences (Piscataway, NJ, USA).

Recombinant SjTGR protein expression and purification was described previously [21]. 1.5 ml of a 100 *μ*g/ml solution of SjTGR in 0.1 M NaHCO3 (pH 8 .6) was added per well to a 12-well plate (Corning Corporation, USA) and incubated at 4°C overnight. Following six washes with Tris-HCL buffered saline (50 mM Tris, 150 mM NaCl, pH 7.5) containing 0.1% Tween-20 (TBST), the well was blocked with 400 *μ*l 5 mg/ml Bovine Serum Albumin (BSA) in Tris-HCL buffered saline (TBS) and incubated for 1 hour (h) at 4°C. Then, 2 x 10^11^ pfu of the phages was added for 1 h at room temperature (rt) with shaking. After six washes with TBST, the bound phages were eluted with 0.5 mM NADPH and 1 *μ*l of phage solution diluted with LB medium was used to determine the phage titer. The remaining eluate was amplified by infecting 20 ml of a 1/100 dilution of an overnight culture of* E. coli* ER2738, as recommended in the instruction manual. The amplified phages were used for the next round of panning, which was repeated twice. The same number of phage particles (2 x 10^11^ pfu) was used in each round. The concentration of Tween-20 for washing was 0.1% for the first panning and 0.5% for the second and third panning.

### 2.2. DNA Sequencing of the Selected Phages Binding with SjTGR

Single phage plaques derived from the third round of panning were amplified and genomic DNA was extracted following the manual. The nucleotide sequences of the inserted peptides (Sangon Corporation, Shanghai, China) were obtained using -96 gIII sequencing primer, 5′-CCTCATAGTTAGCGTAACG-3′, and -28 gIII sequencing primer, 5′-GTATGGGATTTTGCTAAACAAC-3′. The amino acid (aa) sequence was deduced from the nucleotide sequence and compared with DNAman software (Version 6.0).

### 2.3. Phage Binding to Recombinant SjTGR by ELISA

Phage clones were amplified according to the manual. A 96-well plate was coated with 100 *μ*l of SjTGR solution (10 *μ*g/ml, 0.1 M NaHCO3, pH 8.6) overnight at 4°C. After blocking with PBS containing 5% milk powder at rt for 1 h, 100 *μ*l of phage solution with titers of 1 x 10^12^, 2.5 x 10^11^, 6 x 10^10^, 1.5 x 10^10^, and 3.75 x 10^9^ pfu/ml was used per well, respectively. The phage library was set as the negative phage control. Following an incubation at 37°C for 2 h and six washes with TBST (0.5% Tween-20), 200 *μ*l of diluted HRP-conjugated anti-M13 monoclonal antibody solution was added for 1 h at rt. Following six washes with TBST, 100 *μ*l of substrate TMB solution was added for 10 minutes at rt. 50 *μ*l of 2 M sulfuric acid solution (H_2_SO_4_) was added to terminate the reaction and absorbance at 450_nm_ was measured. OD450_nm_ values 2.1 times above the negative control value were considered positive.

### 2.4. Activity of the Isolated Phages and Synthetic Peptides

To assess the inhibitory action of phages, the activities of TrxR, GR, and glutaredoxin (Grx) of SjTGR were assayed as described in [21]. 10 *μ*l (10^14^ pfu/ml) of phage solution was added to the reaction system, phage library was used as negative phage control, and the effect of phages on the activity of SjTGR was expressed as percentage inhibition: Inhibition percentage = (activity of SjTGR – activity of SjTGR with isolated phage)/activity of SjTGR. The experiment was repeated three times.

The activities of TrxR, GR, and Grx of SjTGR were assayed with different concentrations (0.5 *μ*M, 1 *μ*M, and 2 *μ*M) of the synthesized peptide JIPDys1 to determine the 50% inhibitory concentration (IC_50_), and the same amount of PBS was added to the system as control. The experiment was repeated three times. The IC_50_ values were calculated by curve fitting using the SPSS 13.0 software.

### 2.5. Homologous Modeling of SjTGR

The dimer structure of SjTGR was constructed based on the known structure of SmTGR (PDB ID: 2x99 and 2x8c) [26] using software MODELLER 9v8 program [27–29]. Structural refinements were accomplished by energy minimization where the initial 3000 steps of steep descent were followed by 2000 steps of conjugate gradient. Subsequently, 200 ps molecular dynamics was performed to equilibrate the structure at 300K using the software Discovery studio 2.5 (Accelrys, San Diego, USA). The final structure was the three-dimensional structural model of dimer SjTGR.

### 2.6. Computer Simulation of the Conformations of Peptide JIPDys1

The initial three-dimensional structure of peptide JIPDys1 was built by Discovery studio 2.5. The MD calculation was carried out using the CHARMM force field. The SHAKE algorithm was applied to fix all covalent bonds containing a hydrogen atom allowing a 2 fs time step to be used in the integration of Newton's equations. The nonbonded interaction energies and forces were smoothly shifted to zero at 1.2 nm. Before MD simulations were carried out, the peptide of the solvated system was optimized by 2000 steps of steepest descent energy minimization followed by 1000 steps of conjugate gradient energy minimization. After the system was heated up to 300 K, the 10 ns MD simulation at 300 K and 1 atm was carried out. The MD simulation was performed under periodic boundary conditions, and coordinates were saved every 10 ps. The structures of the peptide JIPDys1 during MD simulation were clustered into several different conformations according to RMSD (root mean square deviation).

### 2.7. Peptide JIPDys1 Docking with SjTGR

Different conformations of peptide JIPDys1 from the MD simulation were docked into SjTGR. The docking of the peptide to the SjTGR homology structure was performed using ZDOCK of Discovery studio 2.5, a rigid-body protein-protein docking software [30]. ZDOCK used a fast Fourier transformation to search all possible binding modes for the proteins, performing evaluation based on shape complementarity, desolvation energy, and electrostatics. The top predictions from ZDOCK were then recomputed by RDOCK to improve the energies and eliminate clashes.

### 2.8. Statistical Analysis

All data are given as the mean ± standard deviation (SD). The activities of TrxR, GR, and Grx of SjTGR (absorbance/S (ΔA/S)) with JIPDys1, JIPDys2, JIPDys3, and JIPDys4 were statistically compared with the negative phage control (phage library) using two-tailed Student's t-tests. The activities of TrxR, GR, and Grx of SjTGR (absorbance/S (ΔA/S)) with the synthesized peptide were statistically compared with PBS control using two-tailed Student's t-tests, too. SPSS 13.0 was used for the statistical analyses. Differences between mean values were considered to be significant at the level of 5%.

## 3. Results

### 3.1. Recombinant Phages and Binding to SjTGR by ELISA

Three rounds of biopanning enriched phages that bound well to SjTGR as indicated by the increased recovery (Table 1). Four consensus display peptides, named JIPDys1, JIPDys2, JIPDys3, and JIPDys4, were found in 58 clones of phages randomly selected for sequencing. Their aa sequences are shown in Table 2. Almost half (26 clones out of 58) carried gene sequences identical to JIPDys1.

**Figure 1:**
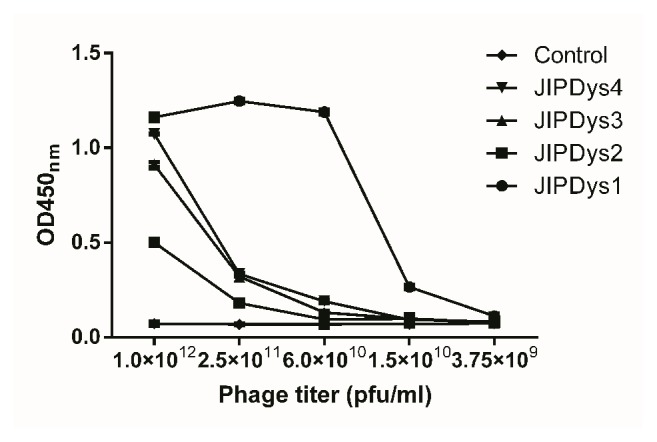
Binding of four selected phages to SjTGR determined by ELISA. The OD450nm values represent the binding ability of JIPDys1, JIPDys2, JIPDys3, and JIPDys4 and the negative phage control (phage library) at five different titers (1.0 x 10^12^, 2.5 x 10^11^, 6.0 x 10^10^, 1.5 x 10^10^, and 3.75 x 10^9^ pfu/ml). Binding decreased with decreasing titers of peptides. Results shown are the mean of triplicates ± SD.

The binding activity of the four peptides was detected by ELISA. The result (Figure 1) showed that at a concentration of 1×10^12^ pfu/ml of all phages, JIPDys1, JIPDys2, JIPDys3, and JIPDys4, bound to SjTGR, although the binding capacity decreased with decreasing concentrations. Phage JIPDys1 displayed the strongest binding capacity among those phages.

### 3.2. Inhibition of SjTGR by Phage Clones

**Figure 2:**
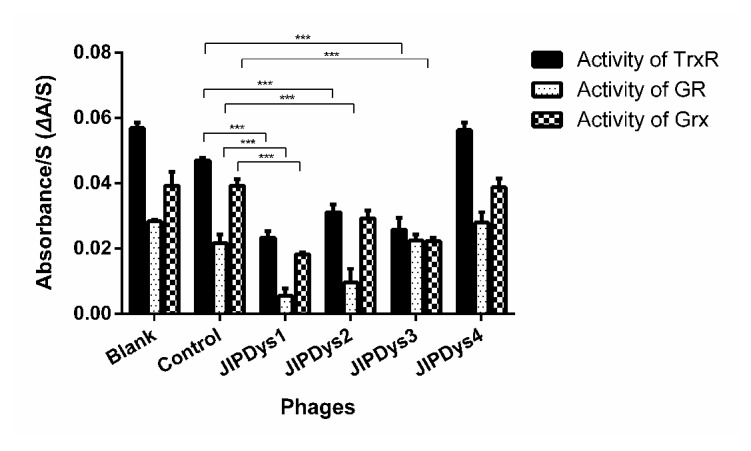
Inhibitory effect of four phages on the activity of TrxR, GR, and Grx of SjTGR. SjTGR was inhibited by 10^11^ pfu of JIPDys1, JIPDys2, and JIPDys3, while JIPDys4 was similar to the negative phage control (phage library). Blank means the activity of TrxR, GR, and Grx without phage. Results shown are the mean of triplicates ± SD. ^*∗∗∗*^The difference was statistically significant (*P*<0.01).

An inhibition test using all four selected phage clones showed that at 10^11^ pfu JIPDys1, JIPDys2, and JIPDys3 inhibited the activities of TrxR, GR, and Grx of SjTGR (Figure 2). The most powerful inhibition of enzyme activity was noted with JIPDys1; the percentages of inhibition of TrxR, GR, and Grx of SjTGR (48 nM) were 59.04%, 80.80%, and 53.56%, respectively. Percentages of inhibition for JIPDys2 were 45.24%, 69.43%, and 25.64%; for JIPDys3 they were 54.72%, 20.88%, and 43.02%; and for JIPDys4 they were 0.73%, 1.50%, and 1.23%, respectively. Comparing the activity of TrxR, GR, and Grx of SjTGR with negative phage control (phage library) and JIPDys1, differences were statistically significant (*P*<0.01). There were statistical differences in the activity of TrxR and GR of SjTGR between JIPDys2 and negative phage control (*P*<0.01). And there were statistical differences in the activity of TrxR and Grx of SjTGR between JIPDys3 and negative phage control (*P*<0.01).

### 3.3. Inhibition of SjTGR by Synthetic Peptide JIPDys1

**Figure 3:**
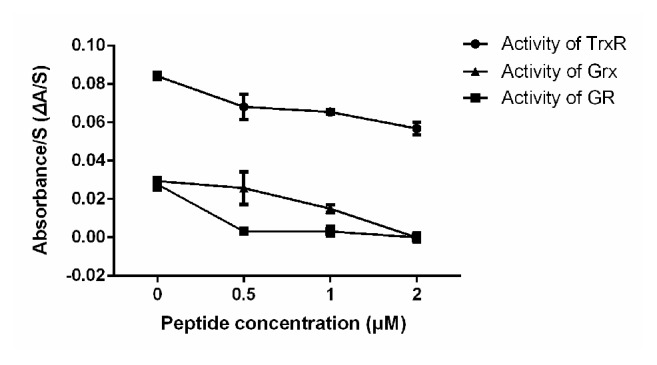
Effect of synthetic peptide on the activity of TrxR, GR, and Grx of SjTGR. SjTGR was inhibited in a concentration dependent manner by the synthesized peptide (0.5 *μ*M, 1 *μ*M, and 2 *μ*M). Results shown are the mean of triplicates ± SD.

As a result of its outstanding performance in initial experiments, the peptide JIPDys1 was synthesized artificially and used as an inhibitor in subsequent experiments. The results showed that 1 *μ*M of the synthesized peptide inhibited the activity of TrxR, GR, and Grx of SjTGR (48 nM) by 22.5%, 88.5%, and 49.1%, and there were statistical differences in the activity of TrxR, GR, and Grx of SjTGR when compared without the synthesized peptide (*P*<0.01). 2 *μ*M of the synthesized peptide inhibited the activity of TrxR, GR, and Grx of SjTGR (48 nM) by 32.5%, 100%, and 100%, respectively (Figure 3), and there were statistical differences in the activity of TrxR, GR, and Grx of SjTGR when compared without the synthesized peptide (*P*<0.01). The IC_50_ values of the synthetic peptide JIPDys1 for TrxR, GR, and Grx were 3.67 *μ*M, 0.11 *μ*M, and 0.97 *μ*M, respectively.

### 3.4. Homologous Structure of SjTGR

**Figure 4:**
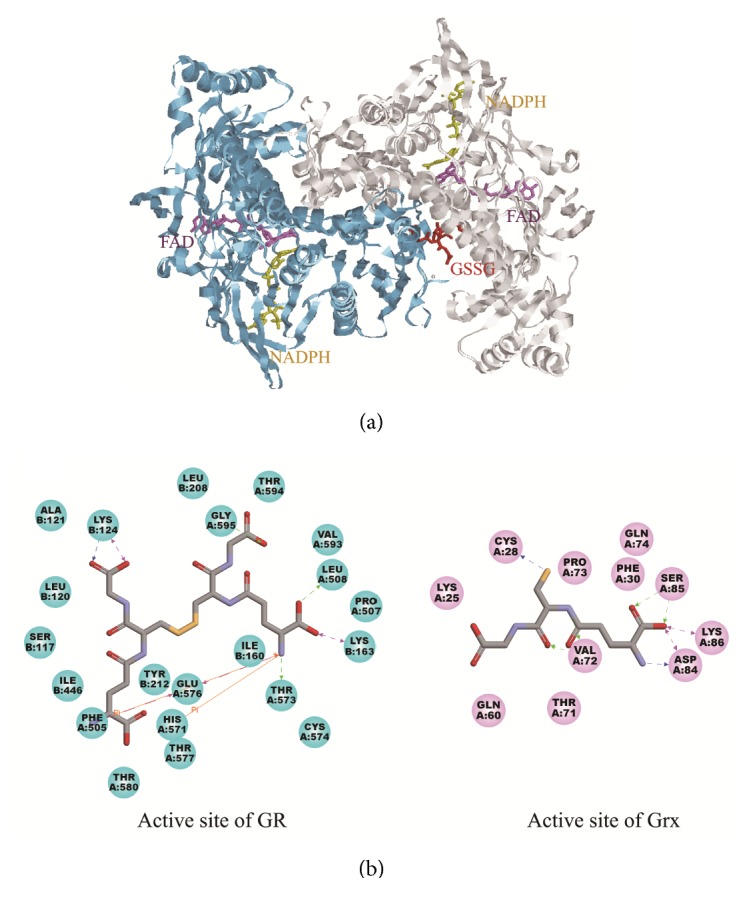
(a) The dimer structure model of SjTGR, including ligands of FAD, NADPH, and GSSG molecules. (b) aa of the GR domain interacting with GSSG (left) and of the Grx domain with GSH (right).

The aa homology of SjTGR and SmTGR was 82% [19]. SmTGR has TrxR and GR function, and its native structure is a homologous dimer. It was used as the template and in accordance with the structure of human oxidized glutathione (GSSG) (PDB ID 2GRT) we set up the dimer model for the SjTGR complex with FAD, NADPH, GSSG, and GSH (Figure 4(a)). The residues of SjTGR interacting with GSSG (the active sites of GR) and GSH (the active sites of Grx) are shown in Figure 4(b). The active site of the GR unit is located at the interface of the dimer, and the residues interacting with GSSG are Ser117, Leu120-Leu 124, Ile160, Leu163, Leu208, Tyr212, and Ile446 of one subunit and Pro507, Leu 508, His 571-Thr 580, and Val 593-Gly 595 of the other subunit. The Grx active domain is N-terminal and its substrate, GSH, is surrounded by Lys25-Phe30, Gln60, Thr71-Gln74, and Asp84-Lys86.

### 3.5. Computer Simulation of Binding between Peptide JIPDys1 and SjTGR

To obtain information on the mechanism of inhibition, interactions of peptide-protein were predicted using the software ZDOCK [29]. Typical conformations of JIPDys1 predicted by MD simulation were selected to simulate docking with SjTGR.

**Figure 5:**
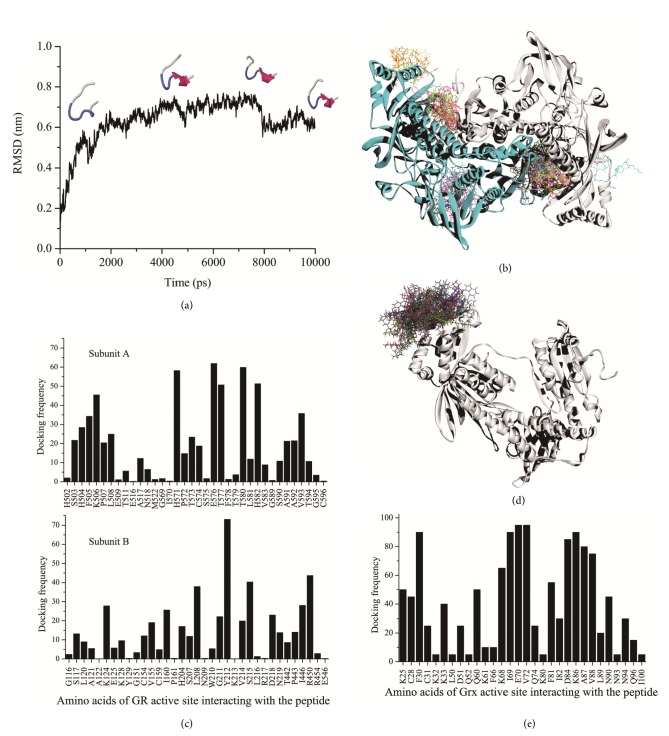
(a) Selected conformations of JIPDys1 during molecular dynamics. (b) Overlap of the peptide JIPDys1 docking with TGR. The regions of SjTGR that interacted with JIPDys1 included the Grx domain, the NADPH binding unit, and the GSSG binding site. The region of high docking frequency centralized mainly at GSSG binding site. (c) Frequency distribution of JIPDys1 docking to the GR active site of SjTGR. (d) Overlap of docked JIPDys1 at the GSH binding site of the Grx domain. (e) Docking frequency of residues at the Grx active site.

Possible structures of JIPDys1 are shown in Figure 5(a). To find suitable binding sites, JIPDys1 was docked into the whole SjTGR. The structures with the highest values (top 10%) were selected for analysis. Figure 5(b) shows that the SjTGR residues that interacted with JIPDys1 were 1-10, 26-30, 45-53, 60-63, 70-83, 124-129, 155, 160, 203-218, 262-255, 293-296, 322-324, 391-394, 420-423, 437-440, 450-463, 501-518, 565-566, and 571-595, including the Grx domain, the NADPH binding site, and the GSSG active site. The region with the highest docking frequencies was identified at the GSSG binding site.

To explore the inhibition of GR activity by JIPDys1 in more detail, the peptide was docked directly into the dimer interface of SjTGR. The frequency of interaction between the residues of the GR domain in SjTGR and JIPDys1 is visualized in Figure 5(c). The values represent the probability of SjTGR residues binding to the peptide. This analysis indicated that the frequency of JIPDys1 interaction with Lys124, Leu208, Tyr212, Ser215, Ile446, and Arg450 of one subunit and Ser503-Leu508, His571, Glu576, Thr577, Thr580, His582, and Val593 of the other subunit of SjTGR was high, suggesting that these residues are most important for the stability of the interaction. In addition, JIPDys1 interacted with Cys154 and Cys159 of SjTGR, although with lower frequency.

Our early work demonstrated that JIPDys1 may inhibit the activity of Grx, and this was supported by simulations which indicated JIPDys1 docking at the GSH binding site of Grx (Figure 5(d)). The result showed high interaction frequencies of JIPDys1 with Phe30, Gln60, Lys68-Val72, and Asp84-Val88 of SjTGR, which are the most important residues for GSH interaction with the Grx domain (Figure 5(e)).

## 4. Discussion

Peptide drugs have aroused general interest for the development of novel drugs due to their ease of usage, fast absorption, and lack of side effects. To date, research exploring peptide drugs has mainly concentrated on tumor treatment, cardiovascular, viral, and microbial diseases, and corneal limbal epithelial stem cell deficiency [31–33]. In addition, development of powerful screening technologies such as phage display library has provided means that have been used in ligand mapping to define peptides that bind to a given antibody or receptor molecule. For example, Sperinde [34] has identified a peptide which could inhibit the activity of DNase II using a circular 12-phage display peptide library. Similarly, Dennis [35] obtained a peptide inhibiting the activity of serine protease by screening a phage display library.

We have previously shown [21] that SjTGR plays an essential role in maintaining the redox balance in* S. japonicum*, which suggested TGR as a potential target for the development of new drugs against schistosomiasis. TGR were detected in the tegument of worms [36]. Peptide drugs against TGR could reach the worm in the blood vessel by intravenous injection. The worm may be dead due to the oxidative damage from the host. Our current work confirms this approach as viable through the identification of a peptide with strong inhibitory activity of SjTGR. Out of the four consensus sequences, JIPDys1, JIPDys2, JIPDys3, and JIPDys4, that were identified by screening a Ph.D.™-12 phage display peptide library, three (JIPDys1, JIPDys2, and JIPDys3) showed inhibitory activity to SjTGR. Among these, JIPDys1 inhibited the activity by more than 50%.

In blast searches using NCBI, the aa sequence of JIPDys1 was 78% identical to the NAD(P) binding region, and JIPDys2 and JIPDys3 were 89% and 64% similar to hypothetical proteins (data not shown). These results suggested that the binding sites and potentially the mechanism of inhibition of JIPDys2 and JIPDys3 were different from those of JIPDys1.

The synthetic peptide JIPDys1 displayed the strongest inhibitory effect on the activity of SjTGR, in a dose dependent fashion. Its inhibitory effect was more pronounced on GR and Grx than TrxR. This is consistent with the results of a molecular docking computer simulation which suggested binding of the peptide to the substrate binding sites of GR and Grx. The region with the highest docking frequencies was identified at the GSSG binding site. Residues in SjTGR that were identified to have high interaction frequency with JIPDys1 are the important aa binding to GSSG. Therefore, JIPDys1 could interfere with GSSG entering into its binding sites, competitively inhibiting the GR activity of SjTGR. The interaction between JIPDys1 and residues of the domain related to TrxR function was not powerful. Although JIPDys1 interacted with Cys154 and Cys159 of SjTGR, the frequency was much lower. Cys154 and Cys159 are related to the electron delivery system of TrxR, and this may be the reason that the inhibitory effect of JIPDys1 on TrxR was not obvious. To enhance the inhibitory effect of the peptide JIPDys1 on the TrxR activity of SjTGR, it would be necessary to remodel the structure of the peptide JIPDys1. This may allow designing a peptide that would inhibit the activity of TrxR, GR, and Grx of SjTGR.

Based on our results one could think of ways to improve the activity of a potential peptide drug by optimizing the aa content of JIPDys1 by adding residues that would bind to the active centers of SjTGR strongly, by reducing hydrophobic aa to increase solubility in water, or by designing novel peptide antagonists of SjTGR by computer simulation and molecular docking taking advantage of the features of JIPDys.

## 5. Conclusion

Taking all together, immune binding, enzyme activity, and computer simulation provide evidence that a novel peptide, JIPDys1 (aa, WPHNWWPHFKVK), could bind with SjTGR and reduce enzyme activity of SjTGR, which is a potential candidate to develop novel drugs against* S. japonicum.* The peptide drugs are easily decomposed by protease* in vivo*, so the structure of peptides needs to be modified by using drug delivery system, which should be studied further. The research makes a foundation for studying peptide drugs against schistosomiasis or provides a new direction for development of the novel drugs against* Schistosoma* infection.

## Figures and Tables

**Table 1:** Enrichment of positive phage clones by panning with SjTGR from Ph.D.-12 phage library.

Rounds	SjTGR (*μ*g/plate)	Phage input (pfu)	Phage Recovery (pfu)	Recovery rate
1	100	1.0 × 10^11^	1.3 × 10^4^	1.3×10^−9^
2	100	1.1 × 10^11^	2.4 × 10^5^	2.2×10^−8^
3	100	2.0 × 10^11^	1.5 × 10^7^	1.7×10^−7^

**Table 2:** Consensus sequences of peptides of recombinant phage clones randomly selected through biopanning from the Ph.D.-12 phage display library.

Phage display peptide	Sequence of peptide	Proportion
JIPDys1	WPHNWWPHFKVK	26 / 58
JIPDys2	LHAETRSAMHRT	2 / 58
JIPDys3	YTMPSLTLYAMG	3 / 58
JIPDys4	KHMHWHPPALNT	4 / 58

## Data Availability

The datasets used and analyzed during the current study are available from the corresponding author on reasonable request.
